# Synergy of antibacterial and antioxidant activities from crude extracts and peptides of selected plant mixture

**DOI:** 10.1186/1472-6882-13-360

**Published:** 2013-12-16

**Authors:** Abdul-Mushin M Shami, Koshy Philip, Sekaran Muniandy

**Affiliations:** 1 Institute of Biological Science, Faculty of Science, University of Malaya, 50603 Kuala Lumpur, Malaysia; 2 Department of Molecular Medicine, Faculty of Medicine, University of Malaya, 50603 Kuala Lumpur, Malaysia

**Keywords:** Indigenous plants, Antibacterial properties, DPPH free radical activity, SOD assay, Pathogenic bacteria

## Abstract

**Background:**

A plant mixture containing indigenous Australian plants was examined for synergistic antimicrobial activity using selected test microorganisms. This study aims to investigate antibacterial activities, antioxidant potential and the content of phenolic compounds in aqueous, ethanolic and peptide extracts of plant mixture*.*

**Methods:**

Well diffusion, minimum inhibitory concentration (MIC) and minimum bactericidal concentration (MBC) assays were used to test antibacterial activity against four pathogenic bacteria namely *Staphylococcus aureus, Escherichia coli, Bacillus cereus,* and *Pseudomonas aeruginosa*. DPPH (2, 2-diphenyl-1- picrylhydrazyl) and superoxide dismutase (SOD) assays were used to evaluate antioxidant activity. HPLC and gel filtration were used for purification of the peptides. Scanning electron microscope was applied to investigate the mode of attachment of the peptides on target microbial membranes.

**Results:**

Aqueous extraction of the mixture showed no inhibition zones against all the test bacteria. Mean diameter of inhibition zones for ethanol extraction of this mixture attained 8.33 mm, 7.33 mm, and 6.33 mm against *S. aureus* at corresponding concentrations of 500, 250 and 125 mg/ml while *E .coli* showed inhibition zones of 9.33 mm, 8.00 mm and 6.66 mm at the same concentrations. *B. cereus* exhibited inhibition zones of 11.33 mm, 10.33 mm and 10.00 mm at concentrations of 500, 250 and 125 mg/ml respectively. The peptide extract demonstrated antibacterial activity against *S. aureus*, *E. coli* and *B. cereus*. The MIC and MBC values for ethanol extracts were determined at 125 mg/ml concentration against *S. aureus* and *E. coli* and *B. cereus* value was 31.5 mg/ml. MIC and MBC values showed that the peptide extract was significantly effective at low concentration of the Australian plant mixture (APM). Phenolic compounds were detected in hot aqueous and ethanolic extracts of the plant mixture. Hot aqueous, ethanol and peptides extracts also exhibited antioxidant activities.

**Conclusions:**

It was concluded that APM possessed good antibacterial and antioxidant activities following extraction with different solvents. The results suggest that APM provide a new source with antibacterial agents and antioxidant activity for nutraceutical or medical applications.

## Background

Australian native herbs have been used in the food industry [1]. These herbs have bioactive compounds such as aldehyde and phenolic compounds that show evidence of antimicrobial activity against microorganisms [2-4]. An Australian plant mixture (APM) that contains four indigenous Australian plants (*Backhousia citriodora*, *Terminalia ferdinandiana*, *Citrus australasica* and *Lophopyrum ponticum* commonly known as Australian wheatgrass sprouts) was examined for synergistic antimicrobial activity using selective test microorganisms. One of these plants is the lemon myrtle or lemon-scented iron wood, *B. citriodora* of the Myrtaceae family. It is found in the subtropical rain forest of central and eastern Australia [5]. While it may grow to 20 m in height, some trees are small. The leaves are opposite lanceolate, evergreen, with an entire margin. The creamy- white flowers are produced in clusters at the end of the branches between summer and autumn [6,7]. *B. citriodora* has been used as a food ingredient, food flavouring agent and herbal tea [8]. It contains essential oils and two main isomeric aldehydes, *cis* and *trans*-citral [9,10]. Hayes and Markovic [11] reported on the antibacterial activity of essential oils of *B. citriodora* against Gram-positive test bacterial strains and Gram-negative test bacterial strains in disc diffusion and MIC assays. The aqueous, ethanol and hexane extracts of *B. citriodora* leaves inhibited the growth of *Enterococcus faecalis*, *P. aeruginosa, E. coli, S. aureus, Salmonella typhi, S. typhimurium and Listeria monocytogenes *[12].

Another plant reported in this mixture is the billy goat plum, kakadu plum or murung, *Terminalia ferdinandiana* of the Combertaceae family. It is a tropical tree found in northern and north western of Australia and grows up to 32 m [13]. It has large, broadly oval, pale green leaves and a flaky bark. The flowers are small and creamy- white and the fruit are yellowish green containing a large seed.

The plant is used as a natural source of vitamin C in dietary health supplements [14]. A high level of total phenolic content is found in kakadu plum fruit reaching a level of 160 μmol gallic acid equivalence GAE/g enhancing its antioxidant capacity along with a high level of vitamin C [15].

The third plant is the finger lime, *Citrus australasica* of the Rutaceae family. It is a small tree, 2–7 m in height, with small leaves and white flowers. The shape of the fruit is cylindrical with different colours such as pink and green. It is found in the southern and northern regions of New South Wales, Australia. It is used in cooking and preparation of jams and drinks [16]. The fruit of finger lime contains a low level of total phenolic content reaching 9.12 μmol gallic acid equivalence (GAE/g) and a small amount of vitamin C so that the antioxidant capacity is relatively low [15]. The essential oils of finger lime inhibited the growth of *S. epidermidis* in electromagnetic field [17].

In addition, APM contains ‘organic’ Australian wheatgrass sprouts claimed to be rich in a wide range of vitamins, minerals, amino acid, and carotenoids.

The aim of this study is to determine the antibacterial and antioxidant activities in hot aqueous, ethanol and peptide extracts of the Australian plant mixture (APM) against *S. aureus*, *B. cereus, E. coli,* and *P. aeruginosa*. This study presents the first report on the antimicrobial activity of hot aqueous, ethanol and peptide extracts of a unique Australian plant mixture that is used as a food supplement. Phenolic content and related antioxidant activity of the hot aqueous and ethanolic extracts were also investigated. It was hypothesized that some of the plant extracts at a certain concentration may display good inhibitory effects against pathogenic bacteria and therefore may aid in the development of antimicrobials and antioxidant supplements.

## Methods

### Extraction procedure

#### *Australian plant mixture (APM) plants and composition*

The mixture (APM) used in the study contains four plants namely *B. citriodora*, *T. ferdinandiana*, *C. australasica* and *L. ponticum* (Table 1). The plants were identified by the Australian Tropical Herbarium where voucher specimens were deposited under reference numbers QRS 34595 for *B. citriodora*, CANB 812161.1 for *T. ferdinandiana*, CANB 673743.1 for *C. australasica* and HO 410713 for *L. ponticum.* The leaves of these plants and sprouts of *L. ponticum* were harvested in October 2010. The leaves and sprouts of the constituent plants in the APM originally sourced from Australia were dried at 40˚C, mixed in the appropriate proportions and extracted as hot aqueous, ethanol and peptide forms.

**Table 1:** Profile contents of Australian plant mixture (APM)

	**Botanical name**	**Common name**	**Family**	**Morphological part of plant used in APM**	**% composition in whole mixture**
1	*Backhousia citriodora*	Lemon myrtle	Myrtaceae	Dried leaves	35% (52.5 g/150 g)
2	*Terminalia ferdinandiana*	Kakadu plum	Combertaceae	Dried leaves	30% (45 g/150 g)
3	*Citrus australasica*	Finger lime	Rutaceae	Dried leaves	20% (30 g/150 g)
4	*Lophopyrum ponticum*	Australian wheatgrass sprouts	Poaceae (syn. Gramineae)	Dried sprouts	15% (22.5 g/150 g)

#### *Hot aqueous extraction*

Five grams of dried powder of this mixture were used for extraction using 50 ml of boiling distilled water for five minutes. After cooling, the mixture was filtered through Whatman No.1 filter paper. The positive control used was 10 mg/ml concentration of tetracycline effective against gram positive and negative microorganisms. Sterile distilled water was used as negative control.

#### *Ethanol extraction*

Five grams of dried powder of this mixture were extracted with 50 ml of 100% of ethanol at room temperature for 3 days with the aid of a magnetic stirrer. The mixture was then filtered through Whatman No.1 filter paper. These liquid was evaporated until the solvent was removed at 40°C using a rotary evaporator (Heidolph WB2000, Germany). The product was purified by using SEP-PAK C18 column. The sample was eluted from the column using 100% ethanol as the mobile phase. The ethanol in the eluate was evaporated to dryness under reduced vacuum at 40°C, weighed and dissolved in 1 ml of 5% Tween 80 to be tested for antibacterial and antioxidant activities.

#### *Peptide extracts from the selected plant mixture*

Ten grams of dried powder of APM was added to a solution of 20 mM of Tris–HCl buffer and polyvinylpyrrolidone (PVP) at a concentration of 20 mg/ml. This method was based on [18] with some modification. This mixture was filtered and centrifuged at 4500 g for 30 minutes at 4°C. The supernatant was removed and precipitated with gradually saturated ammonium sulphate at 4°C overnight. The supernatant was separated by centrifuging at 4500 g for 30 minutes at 4°C, and the resulting pellet was collected and tested for antibacterial and antioxidant activities.

#### *Total protein estimation*

The samples were measured for total protein concentration by Bradford method [19]. Briefly, 100 μl of the samples were added to 3 ml of Bradford reagent (containing Coomassei Brilliant Blue G-250) and then incubated at room temperature for 15 min. Absorbance of the samples were measured at 595 nm. Bovine serum albumin at different concentrations was used to plot a standard curve to calibrate protein concentration of the extracts as a measure of the peptide amount.

### Antibacterial assays

#### *Well diffusion assay*

In this study, four bacterial test strains were used namely *S. aureus* (RF 122), *E. coli* (UT181), *B. cereus* (ATTC 14579) and *P. aeruginosa* (PA7) isolated from University of Malaya hospital and deposited in our laboratory type culture collection strains. These bacteria were inoculated into Mueller-Hinton agar (Difco, Detroit, MI, USA) using a swab. The bacteria was inoculated into this medium at 1 × 10^8^ CFU/ml concentration based on optical density (O.D.) measurement at 620 nm.

Wells of 6 mm in diameter and depth were made on the media plates. All extracts were dispensed into the wells (50 μl), and incubated overnight at 37°C. Inhibition zone assays using well diffusion were conducted in triplicate. The positive control used was Tetracycline (10 mg/ml) and the negative control comprised 5% Tween 80 aqueous solution.

#### *Minimum inhibitory concentration (MIC) and minimum bactericidal concentration (MBC)*

Aqueous, ethanolic and peptides extracts of APM were determined for their MIC values using a standard protocol [20]. Nutrient broth (Difco, Detroit, MI, USA) was used as the medium to culture bacteria. One ml of this broth was added to the numbered tubes 1–9. One ml of the stock culture was added to tube 1 and serially diluted until tube number 7. The last 1 ml of tube 7 was discarded. Tube number 8 was used as a negative control and the tube 9 as a positive control. The bacterial inoculum was cultured in nutrient broth and incubated overnight. All the tubes were inoculated with 1 ml of the test bacteria media except tube number 8 and incubated for 24 hrs at 37°C. MIC values were determined based on the minimum inhibition observed in the tube that showed no growth. MBC values were determined by sub-culturing from the MIC assay tubes onto Muller- Hinton agar (Difco, Detroit, MI, USA) and then determining the dilution at which growth was detected. McFarland standard (0.5) was used to determine the amount of colony forming units (CFU) of the bacteria in nutrient broth (1 × 10^8^ CFU/ml) based on optical density (O.D.) measurement at 620 nm.

### Antioxidant assays

#### *DPPH radical scavenging assay*

Free radical scavenging activity of the hot aqueous, ethanol and peptide extracts of APM were determined by using the method of Bozin et al. [21] with some modifications including the amount of samples (10 mg/ml) and conditions of incubation (dark, 25°C for 2 hrs). The reagent of the assay is 2, 2- diphenyl-1- picrylhydrazyl solution (Sigma Aldrich GmbdH, Germany) (950 μl) that was added to 50 μl of the extract (10 mg/ml) and the volumes of the solutions made up to 4 ml by adding 95% ethanol. This mixture was shaken vigorously and incubated at room temperature for two hours in the dark. All samples were measured at 515 nm using a Genesys 20 Thermoscientific (USA) spectrophotometer. The percentage of DPPH radical scavenging activity of the resulting solutions was calculated by using the following equation:

$$\begin{array}{l} \mathrm{DPPH}\;\text{radical}\;\text{scavenging}\;\text{activity}\;\left(\%\right)\\ \;=\left[(A_{\text{control}}–A_{\text{sample})}/A_{\text{control}}\right]\;x100 \end{array}$$

Ascorbic acid (10 mg/ ml) was used as a positive control of the assay.

IC_50_ was calculated using linear regression plots. The IC_50_ values represent the concentrations of the sample that is required to scavenge 50% of DPPH free radicals.

#### *SOD assay*

SOD activity was determined using a SOD Assay kit-WST (Dojindo Molecular Technologies, Gaithersburg). The samples (20 μl) were mixed using the reaction mixture in the kit. Then, the mixtures were gently shaken and incubated at 37°C for 20 min. Antioxidant activity was measured at 450 nm using a Genesys 20 Thermoscientific (USA) spectrophotometer. The positive control used was ascorbic acid (10 mg ml^-1^). The protocol used in this study was modified from [22]. The modifications included varying the amount of samples used and the incubation period. The negative control to measure inhibition rates of SOD activity used all treatments without sample.

#### *Total phenolic contents*

Total phenolic contents of the hot aqueous and ethanol extracts of APM were determined by Folin- Ciocalteau method based on [23]. Briefly, 20 μl of the samples were added to 100 μl of 2 N Folin- Ciocalteau reagent. The final volume of the mixture was adjusted to 1600 μl using distilled water. After that 300 μl of 10% of sodium carbonate was added and incubated at 37°C for 45 minutes. These solutions were measured using a Genesys 20 Thermoscientific (USA) spectrophotometer at 760 nm wavelength. Gallic acid at concentrations of 50 to 1000 mg/ml was used to plot a standard curve to calibrate total phenolic contents in the extracts.

### Peptide purification

#### *Purification of peptides from selected plants mixture by gel filtration and HPLC*

The crude peptides extracts from APM were purified by sephadex G-75 (Sigma). The freeze-dried sample was loaded onto a column of dimension 1.5 × 73 cm. It was eluted with 20 mM Tris-buffer, pH 7.6. The flow rate was maintained at 1.5 ml/5 min. The fractions were collected and measured at 280 nm. All fractions were tested for antibacterial activity. The active fraction (F1) was dissolved 0.1% aqueous trifluoroacetic acid (TFA) solution (3 ml) and fractionated by reverse phase-HPLC (Agilent 1100 series) with Zorbax 300SB-C18 (4.5 × 150 μm, 50 μm, 300A°) at a flow rate of 1.0 ml/min. The solvents used were: (A) 0.1% TFA in water and (B) 0.1% TFA in acetonitrile. A step gradient of solvent A was used to run the column as follows: 0-60% for 0–45 min, 60-80% for 45–55 min and 80-100% for 55–60 min. The fractions of the mixture were detected at 245, 215 and 280 nm. Active fraction of APM was collected at 4.181 min and then lyophilized and stored at -20°C.

#### *LC-MS/MS*

LC-MS analysis was conducted using active fraction of APM that was rehydrated to 500 μL of 2% acetonitrile with 0.1%TFA centrifuged for 3 min at 13,000 rpm. The chromatography system was coupled to an LTQ Orbitrap Velos mass spectrometer equipped with a Nanospray II source (Thermo Fisher Scientific). Solvents were A: 2% acetonitrile, 0.1% formic acid; B: 90% Acetonitrile, 0.1% Formic acid. After a 249 bar (~5 μL) pre-column equilibration and 249 bar (~8 μL) nanocolumn equilibration, samples were separated by a 55 minute gradient for IT-CID method (0 min: 5%B; 45 min: 45%B; 2 min: 95%B; 8 min: 95%B) or 70 minute gradient for FT-CID method (0 min: 5%B; 60 min: 40%B; 2 min: 80%B; 8 min: 80%B). The LTQ Orbitrap Velos parameters were as follows: Nano-electrospray ion source with spray voltage 2.2 kV, capillary at temperature 225°C. Survey MS1 scan *m/z* range 400–2000 profile mode, resolution 60,000 or 30,000 -*400 m/z* with AGC target 1E6, and one microscan with maximum inject time 200 ms.

Raw files were analysed with Proteome Discoverer 1.3.0.339 software suite (Thermo Scientific). The peak lists were submitted to an in-house Mascot 2.2 against the Uniprot-Swissprot. 20110104 (523,151 sequences; 184,678,199 residues) database as follows: precursor tolerance 8 ppm; MS/MS tolerance 0.6 Da (Iontrap), 0.020 Da (FT); No enzyme; FT-ICR ESI instrument type; variable modifications: deamidation (N,Q); oxidation (M). Percolator settings: Max delta Cn 0.05; Target FDR strict 0.01, Target FDR relaxed 0.05 with validation based on q-Value.

### Mode of action

#### *Effect of peptide extracts from APM by scanning electron microscope*

Bacterial culture (*B. cereus*) was incubated in nutrient broth overnight at 37°C. This culture (1 ml) was added to one milliliter of bioactive fraction of peptide extracts of APM and kept for 4 hr at 37°C. This mixture was then centrifuged at 6500 g at 4°C for 10 min. The pellet was washed twice with 50 mM sodium phosphate buffer (pH 7). The bacterial cells were re-suspended with buffer and 1 μl of suspension deposited on a membrane filter. Bacterial cells were fixed with 8% glutaraldehyde for 1 hr. The fixed cells were washed with buffer in distilled water in a ratio of 1:3 for 15 min. The bacterial cells were dehydrated in ascending concentrations of ethanol (10, 20, 30, 40, 50, 60, 70, 80, 90, 95, 100 and 100%) at 15 min exposure for each concentration. The bacterial cells were further dehydrated in different ratios of ethanol: acetone (3:1, 1:1 and 1:3) for 20 min for each mixture and then washed with pure acetone four times each for 20 min. These bacterial cells were subjected to critical point drying using liquid CO_2_ and the cells mounted on a stub. These cells were coated with gold and examined by using scanning electron microscope (Model: JEOL JBM 7001 F, UK). The control used for this experiment is normal, untreated bacterial cells, compared to *B. cereus* treated with peptide extracted from APM (F1).

### Statistical analysis

Data is expressed as mean ± SD. Statistical analyses were performed using SPSS version 17. One-way ANOVA followed by Duncan’s multiple comparison was used to compare the values of samples with control. A *P* value < 0.05 was regarded as indicating significant differences. Each treatment was replicated 3 times and each experiment was repeated at least twice.

## Results

### *Antibacterial activity*

**Figure 1:**
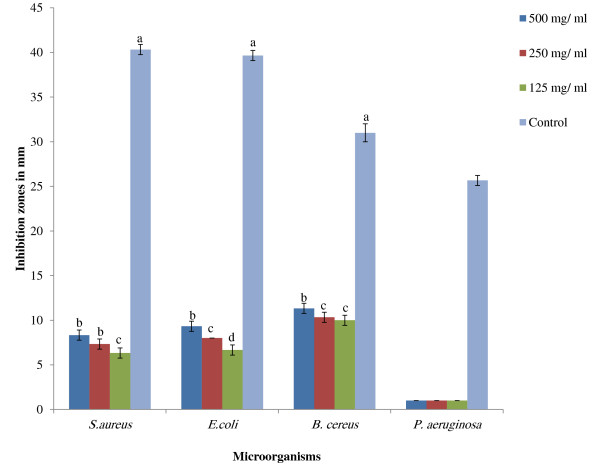
**Inhibition zones of ethanolic extract of selected plant mixture on selected test microorganisms.** Different letters **(a- d)** showed significant difference (*p* < 0.05) from all samples and control (10 mg/ml of Tetracycline) by using one-way ANOVA followed by Duncan’s Multiple Comparison. *All samples in three replicates ± SD.

**Figure 2:**
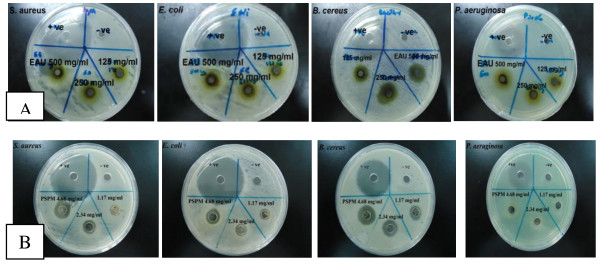
**Inhibition zones of different extracts of selected plant mixture on selected test microorganisms. (A)** effect of ethanolic extract of selected plant mixture (EAU) and **(B)** peptides extracts (PAUS) against selected test microorganisms with different concentration compare to positive control (10 mg/ ml of Tetracycline).

**Figure 3:**
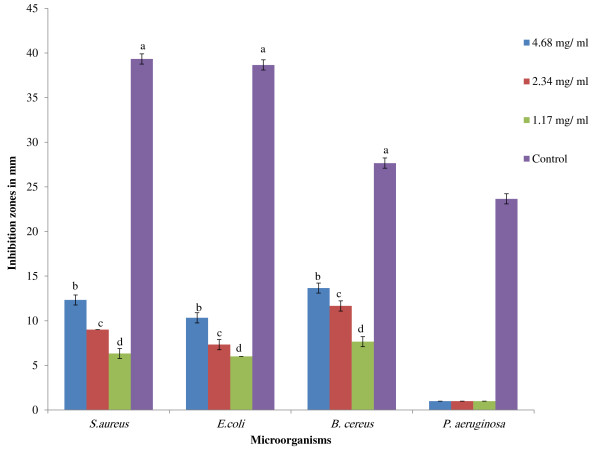
**Inhibition zones of peptide extract of selected plant mixture on selected test microorganisms.** Different letters **(a- d)** showed significant difference (*p* < 0.05) from all samples and control (10 mg/ml of Tetracycline) by using one-way ANOVA followed by Duncan’s Multiple Comparison. *All samples in three replicates ± SD.

**Figure 4:**
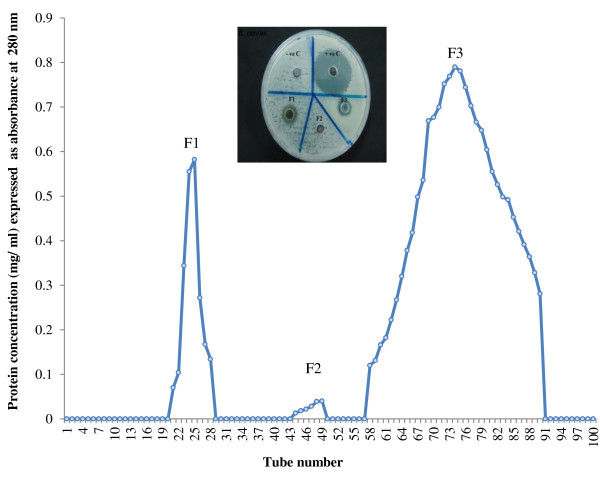
Fractionation of peptides extracted from selected plant mixture by sephadex G-75 gel filtration.

The results of the hot aqueous extract of APM showed no inhibition zones on all the test microorganisms, namely *S. aureus*, *E. coli*, *B. cereus,* and *P. aeruginosa* by well diffusion assay. Figures 1 and 2 show zones of inhibition of the ethanolic extract of the mixture ranging from 8.33 mm ± 0.57 for *S. aureus* to 9.33 mm ± 0.50 for *E. coli* and from 11.33 mm ± 0.57 for *B. cereus* at high concentration of this extract (500 mg/ ml) but no inhibition by *P. aeruginosa*. It is noteworthy that *B. cereus* was most sensitive of the test bacteria to the ethanol extract, being sensitive at a concentration of 50 mg/ml. On the other hand, peptides extract showed zones of inhibition against *S. aureus*, *E. coli* and *B. cereus* at a lower concentration of 1.17 mg/ml (Figures 3 and 2). MIC and MBC values for hot aqueous extract of the mixture showed no result on all test bacteria, but the ethanolic extract showed minimum inhibition at 125 mg/ml for *S. aureus* and *E. coli* while *B. cereus* at 31.5 mg/ml. Again, no inhibition result was obtained for *P. aeruginosa* (Table 2). However, MIC and MBC values of crude peptide extract of the mixture was obtained at 1.17 mg/ml for *S. aureus* and *E. coli* with *B. cereus* being inhibited at a lower concentration of 0.58 mg/ml (Table 2). Figure 4 shows that the fraction 1 of APM had higher antibacterial activity against *B. cereus* compared to *S. aureus* and *E. coli*.

**Table 2:** MIC and MBC of ethanolic and peptides extracts of plant mixture (APM) against selected test microorganisms

**Bacteria**	**Plant extracts (mg/ml)**			
	**Ethanolic extract**		**Peptide extract**	
	**MIC**	**MBC**	**MIC**	**MBC**
***S. aureus*** (RF 122)	>125	125	1.17	>1.17
***E. coli*** (UT181)	125	125	1.17	<1.17
***B. cereus*** (ATTC 14579)	31.25	>31.25	0.58	>0.58
***P. aeruginosa*** (PA7)	0.00	0.00	0.00	0.00

### Antioxidant activity

**Figure 5:**
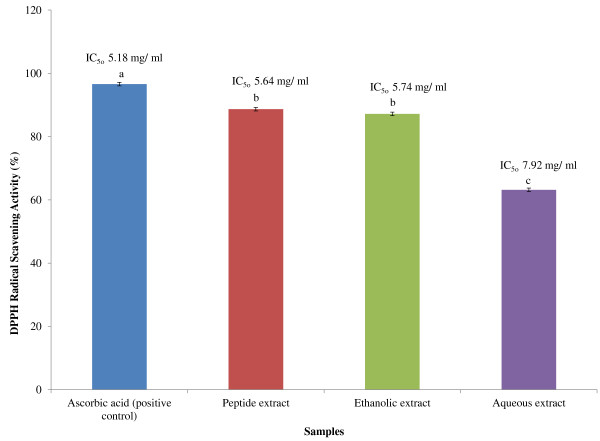
**DPPH scavenging activity of different extracts of selected plant mixture.** The values are the average of three replicates ± SD. The results were analyzed by one-way ANOVA followed by Duncan’s Multiple comparison Test. Different letters **(a- c)** showed significant difference (*p* < 0.05) from all samples and control. Positive control indicates to 10 mg/ml of ascorbic acid.

**Figure 6:**
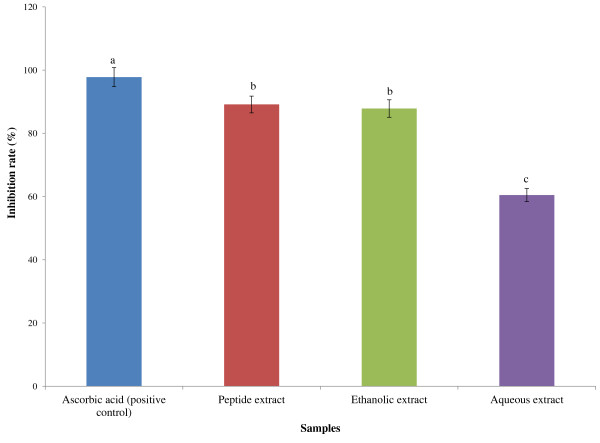
**Inhibition rate of SOD activity of different extracts of selected plant mixture.** The results were analysed by one-way ANOVA followed by Duncan’s Multiple comparison Test. Different letters **(a- c)** showed significantly difference (p < 0.05) from all samples and control. Positive control used 10 mg/ml concentration of ascorbic acid.

Figure 5 shows the percentage of DPPH radical scavenging activity of the hot aqueous extract at 63.19% (IC_50_ 7.92 mg/ml), ethanolic extract at 87.17% (IC_50_ 5.74 mg/ ml), peptide extract at 88.66% (IC_50_ 5.64 mg/ml) compared to ascorbic acid as a positive control at 96.59% (IC_50_ 5.18 mg/ml). Inhibition rate of SOD activity of hot aqueous extracts of the mixture was 60.5% but ethanolic extract reached 87.87%. However, peptide extract showed significant antioxidant activity at 89.16% (Figure 6). The determination of total phenolic content in the APM showed the highest amounts of phenolic compounds in the ethanolic extract at 427.70 mg GAE/g while the hot aqueous extract was at 283.12 mg GAE/g (Table 3).

**Table 3:** Total phenolic content of Australian Plant Mixture extracts (APM)

**Concentration**	**Total phenolic content (mg GAE/g)**	
	**Ethanolic extract**	**Hot aqueous extract**
100 mg/ml	427.70 ± 2.60^a^	283.12 ± 1.25^a^
50 mg/ml	248.53 ± 1.90^b^	169.37 ± 3.75^b^
10 mg/ml	93.95 ± 0.75^c^	57.74 ± 1.40^c^

The values are the average of triplicates ± SD. The results were analysed by one-way ANOVA followed by Duncan’s Multiple Comparison Test. Different letters (a - c) showed significant difference (*p* < 0.05) for all samples and control.

### Attachment of peptide extracts to *B. cereus* cells as examined under SEM and LC- MS/MS analysis

**Figure 7:**
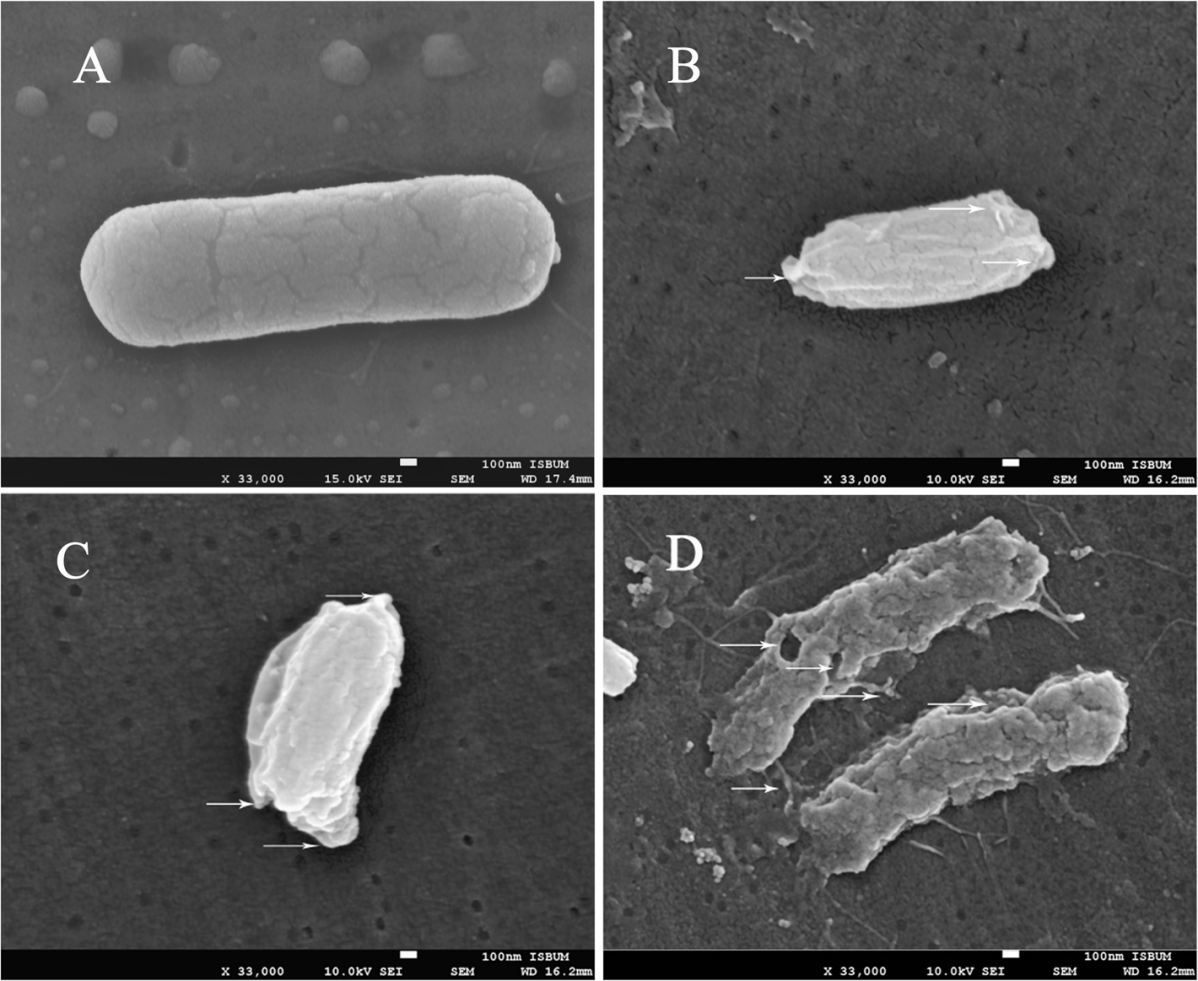
**Effect of peptides extracted from Australian plant mixture (F1) by scanning electron microscope. (A)** and **(C)** control: *B. cereus.***(B)** and **(D)***B. cereus* treated with peptides.

**Figure 8:**
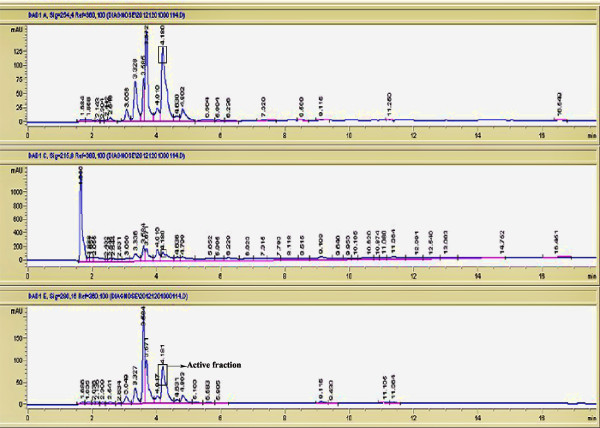
**HPLC chromatogram of F1 of selected plant mixture detected at 254, 215 and 280 nm.** Active fraction peak was at 4.181 min.

**Figure 9:**
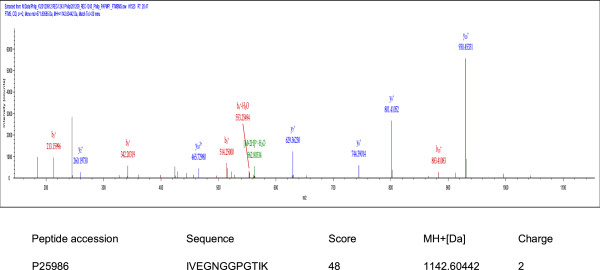
LC - MS/MS analysis of active fraction of APM with amino acid sequences.

Figure 7 shows the effect of peptides from APM against *B. cereus.* It was noted the bacterial cells treated with antibacterial peptide (Fraction 1) of the mixture displayed significant modifications in cell morphology and the shape. These modifications included roughening in cell surface, numerous blebs, and lysis of bacterial cells and accumulation of cell debris. The bioactive fraction 1 fractionated by HPLC gave several peaks on further profile purification. The active fraction of APM peaked at 4.181 min inhibitory to all test bacteria except *P. aeruginosa* (Figure 8). LC- MS/MS analysis of active fraction of APM showed the amino acid sequences (IVEGNGGPGTIK) after referring to Uniprot-Swissprot software to determine the amino acid sequences (Figure 9). The name of the peptide was Pathogenesis-related protein 2 OS = Phaseolus vulgaris.

## Discussion

The results from this study reveal that APM has antibacterial and antioxidant activities. The hot aqueous extract showed no antibacterial activity perhaps because the concentrations of bioactive compounds in this extract were low. In addition, the active compounds were soluble in organic solvents [24]. Ethanolic extract of the mixture had antibacterial activity against selected Gram-positive bacteria (*S. aureus*, *B. cereus*) and Gram-negative bacteria (*E. coli*). Past studies have reported the essential oils in *B. citriodora* showing antibacterial activity against *S. aureus, E. coli,* methicillin- resistant *S. aureus* (MRSA), *Aspergillus niger, Klebsiella pneumoniae,* and *Propionibacterium acnes *[11]. Previous studies have documented that leaf paste of *B. citriodora* shows antibacterial activity against *Clostridium perfringens, P. aeruginosa,* MRSA, molds and fungi [25].

Another plant in the mixture is finger lime, *C. australasica*. It has essential oils with antibacterial effect against *S. epidermidis* in an electromagnetic field [17]. In addition, *T. ferdinandiana* contains a high level of phenolic compounds. These substances have been demonstrated to show antibacterial activity against microorganisms [26,27]. MIC and MBC values of ethanolic extracts of this mixture showed that the extract is very potent against the selected test bacteria with *B. cereus* being very sensitive. These results were in agreement with the work of Dupont et al. [12] that found significant values of MIC when used against *E. faecalis*, *P. aeruginosa, E. coli, S. aureus, S. typhi, S. typhimurium,* and *L. monocytogenes* in aqueous, ethanol and hexane extracts of these Australian native herbs. However, *S. aureus* was more sensitive. MIC results of Australian essential oil of *B. citriodora* revealed antibacterial and antifungal activities against *S. aureus, E. coli,* MRSA, *A. niger, K. pneumoniae* and *P. acnes *[11]. Peptide extracts of APM have antibacterial activity because of their ability to disrupt the membrane of bacteria by interacting with lipid molecules on the cell surface of *B. cereus *[28].

Results of the current study show that ethanolic and peptide extracts of APM have a high percentage of DPPH radical scavenging activity with significant IC_50_ values compared to hot aqueous extract. Therefore the ethanolic and peptide extracts have good antioxidant activity. In addition, the ethanolic and peptide extracts have a high level of inhibition rate for SOD activity. Earlier studies show these compounds have hydroxyl groups in aromatic rings of the structure that are able to reduce agents by hydrogen -donating antioxidant and singlet oxygen quenching actions [29-31] . The mode action of antibacterial peptides of APM appear to follow a membrane disruption model because peptide- mediated membrane disruption depended on the peptide integrity of cell membrane or peptide translocation into the cytoplasm [31,32]. Fraction 1 of APM showed antibacterial activity against all bacteria except *P. aeruginosa* but was more sensitive to *B. cereus.* LC-MS/MS results showed active fraction that have sequences of amino acids with antibacterial activity against pathogenic bacteria (Figure 9). Wong et al. [33] reported this peptide (defensin) to have antibacterial activity against *Mycobacterium phlei, Bacillus megaterium, Bacillus subtilis and Proteus vulgaris* because this peptide has cysteine residue in the structure. Also it has antifungal activity against *Botrytis cinerea*, *Fusarium oxysporum* and *Mycosphaerella arachidicola*. Defensin also has antifungal properties against *Mycosphaerella arachidicola*, *Setospaeria turcica* and *Bipolaris maydis *[34] . The results above strongly suggest that the antioxidant and antibacterial properties of APM are due to synergistic effect of the individual components.

## Conclusions

In conclusion, this is the first report that studied antibacterial activity and antioxidant capacity in hot aqueous, ethanolic and peptide extracts of the APM that is consumed as a natural food blend. While the hot aqueous extract showed no effect against all test bacteria strains in well diffusion, MIC and MBC assays, the ethanolic and peptide extracts had good antibacterial activity against *S. aureus, E. coli* and *B. cereus*. However, no positive result was obtained for *P. aeruginosa* using the different assay techniques in this study. All extracts of the mixture showed antioxidant ability against DPPH free radicals and SOD assays. Further studies are ongoing to isolate and identify bioactive compounds in the mixture that may be used to develop biopharmaceuticals against infectious diseases in future.

## Competing interests

The authors declare that they have no competing interests.

## Authors’ contributions

AMS carried out the plant extractions, antibacterial and antioxidant experiments. KP supervised the research work protocols and prepared the manuscript. SM evaluated the data and read the manuscript. All authors read and approved the final manuscript.

## Pre-publication history

The pre-publication history for this paper can be accessed here:

http://www.biomedcentral.com/1472-6882/13/360/prepub
